# CBX3 promotes clear cell renal carcinoma through PI3K/AKT activation and aberrant immunity

**DOI:** 10.1186/s12967-023-04478-9

**Published:** 2023-09-06

**Authors:** Jiasheng Chen, Yuxin Lin, Shukai zheng, Qingshan Chen, Shijie Tang, Xiaoping Zhong

**Affiliations:** Department of Burns and Plastic Surgery, The Second Affiliated Hospital of Shantou University Medical College, Shantou, Guangdong People’s Republic of China

**Keywords:** Clear cell renal cell carcinoma, CBX3, Progression, PI3K/AKT signaling pathway, Immunologic disorder

## Abstract

**Background:**

A chromobox homologue 3 (CBX3) is elevated in various cancers and significantly contributes to the promotion of malignant behavior; despite this, its exact involvement in clear cell renal cell carcinoma (ccRCC) is yet unknown.

**Methods:**

The Cancer Genome Atlas database served to evaluate CBX3 production and its connection to survival in patients with ccRCC. Our team evaluated the effects of knockdown of CBX3 levels in ccRCC cell populations using in vitro together with in vivo models. CBX3, proteins related to death, and epithelial-to-mesenchymal transition (EMT)-related proteins were measured in ccRCC cells using western blotting and immunohistochemical assays. Through the analysis of Kyoto Encyclopedia of Genes and Genomes (KEGG) and GeneOntology (GO) and Gene Set Enrichment Analysis (GSEA), the biological processes and signal pathways related to CBX3 expression were identified. Immune-related activity reduced by CBX3 was assessed using various online tools.

**Results:**

Both genomic and protein expression showed that CBX3 was upregulated in ccRCC. Further functional analyses revealed that CBX3 played a crucial role in enhancing cell growth, migration, and EMT in vitro along with in vivo. Moreover, the study results provided distinct mechanistic evidence that CBX3 exerts its pathological functions in ccRCC by activating the PI3K/AKT pathway. Finally, immunoassays revealed that CBX3, a possible biomarker of ccRCC, was significantly associated with immunity.

**Conclusions:**

Our results suggest that the overexpression of CBX3 promotes ccRCC advancement through PI3K/AKT activation and even immunological dysregulation, making it a potentially viable and beneficial therapeutic target.

**Supplementary Information:**

The online version contains supplementary material available at 10.1186/s12967-023-04478-9.

## Introduction

Kidney cancer is considered one of the leading ten causes of cancer-associated deaths, and clear cell renal cell carcinoma (ccRCC) is the most common subtype [1, 2].Owing to the insidious and asymptomatic nature of early-stage RCC, many patients already exhibit metastasis at diagnosis [3]. In the advanced stages, traditional modalities, such as surgery, chemotherapy, and radiotherapy, are ineffective for ccRCC [4, 5]. Therefore, early detection, diagnosis, and treatment are key to improving the future prospects of individuals with ccRCC. As a result, there is a pressing need to develop superior targets for diagnosis and treatment of ccRCC.

CBX3, a chromobox homologue 3, is also known as heterochromatin-associated protein 1 γ (HP1γ) and is a highly conserved non-histone chromosomal protein. As a major component of heterochromatin, its chromatin domain can bind to methylated histone H3K9, thereby recruiting various cofactors or target proteins to participate in the negative regulation of transcription by binding to DNA [4, 5]. CBX3 controls biological functions such as DNA repair, gene silencing, and cell division and differentiation [7, 8]. Recent studies have shown that CBX3 overexpression promotes tumorigenesis and progression in numerous malignancies, especially hepatocellular carcinoma, glioblastoma, and colon cancer [9–12]. CBX3 could potentially serve as a predictive biomarker for ccRCC [9–12], and its significant association with the infiltration level of immune cells are apparent; however, the precise molecular mechanisms by which CBX3 accelerates the development of ccRCC remain elusive.

The PI3K/AKT pathway’s association with tumor progression is well documented. Several studies have highlighted the overactivation of the PI3K/AKT [14], which is linked to poorer clinical outcomes in patients with RCC [15]. Additionally, the potential for CBX3 to influence prostate cancer growth by modulating the PI3K/AKT pathway has been suggested [16]; however, the significance of the interplay of CBX3 with the PI3K/AKT pathway in ccRCC is unclear.

The tumor microenvironment (TME) is a dynamically complex multicellular environment that influences tumor development [17]. According to previous reports, changes in the immune cell ratio in the TME of RCCs can impact both patient prognosis and therapeutic efficacy. The tumor genome may also affect the TME  to present variable patterns in ccRCC [18]. Numerous studies have demonstrated the connection between CBX3 and immune infiltrating cells, immunosurveillance sites, immune components, and stromal cells in ccRCC [13, 19]; nevertheless, the function of CBX3 in ccRCC immunization remains poorly understood.

Therefore, we aimed to investigate the function of CBX3 in ccRCC by examining CBX3 expression, conducting survival analysis and functional in vitro and in vivo assays, investigating the relationship between CBX3 and potential underlying mechanisms, and conducting comprehensive immunoassays and potential drug analysis. This study contributes to the growing pool of evidence highlighting the significance of CBX3 as a predictive biomarker and therapeutic focus for ccRCC.

## Materials and methods

### Data acquisition and preprocessing

RNA sequence expression profiles, phenotype information, and survival information in the Genomic Data Commons Cancer Genome Atlas Kidney Clear Cell Carcinoma (TCGA-KIRC) dataset were accessed using the University of California Santa Cruz Xena Browser (https://xenabrowser.net). The dataset was then processed and 606 samples were obtained, including 534 KIRC and 72 normal samples.

### Analysis of CBX3 expression levels and prognosis in KIRC

Differences in the expressabilty of CBX3 in KIRC and matched normal tissues were first investigated. To identify characteristics of CBX3 in renal cancer cell lines, the mRNA expression matrix of renal cancer cell lines was obtained from the Cancer Cell Line Encyclopedia (CCLE) dataset (https://portals.broadinstitute.org/ccle). Next, the relationship between CBX3 expression levels and the prognosis of KIRC patient was evaluated and visualized by the Kaplan–Meier curve. All patients were grouped into different pathological stages including T1, T2, T3, T4, N0, N1, NX, M0, M1, MX,T1 + T2 and T3 + T4. These groups were further subdivided based on high- and low-expression levels of CBX3. Subsequently, hazard ratios and 95% confidence intervals (95% CI) were calculated, and a log-rank test was conducted.

### Cell cultivation

The kidney cancer cell lines (ACHN, 786-O, OS-RC-2, and 769-P) as well as the HK-2 cell line were obtained from the American Type Culture Collection (ATCC, Manassas, VA, United States). These cell populations were cultivated in RPMI-1640 medium (Gibco, Carlsbad, USA) with the addition of 10% fetal bovine serum (Gibco, Carlsbad, USA), 100 mg/mL streptomycin sulphate, and 100 μL/mL penicillin (Gibco, Carlsbad, USA).

### Cell transfection

The 769-P and ACHN cell lines were seeded overnight in 6-well plates as well as transfected with shRNA (Hanbio Biotechnology Co., Ltd, Shanghai, China) at 60% Lipofectamine 3000 (Invitrogen CA, USA) following the manufacturer's instructions. After 48 h of subincubation, the cells were harvested and applied to further experiments.

### Cellular proliferation assays

Cells were seeded overnight at 1 × 10^3^ cells/well in 96-well plates, and 10 µL of Cell Count Kit-8 solution (Beyotime Biotechnology, Jiangsu, China) was applied to each well and incubated for 2 h at 37 °C. Cell survival was assessed at the end of each time point (0, 24, 48, 72 h). At 540 nm after incubation, absorbance was measured.

### Colony formation assays

Cells were cultured at 37 ℃ for 14 d at a total of 600 cells/well in a 6-well plate. Cell fixation was performed using 4% paraformaldehyde and staining with crystal violet (0.5%). Colonies were then counted and photographed.

### Wound healing assays

Cell migration was assessed by seeding 5 × 10^5^ cells/well in a 24-well plate, which was cultured at 37 ℃ until the cell density reached 80% confluence. An artificial wound was created using a sterile pipette tip of 10 µL, and the cells were then cultivated in serum-free medium. An inverted microscope (Olympus Corp. TKY, JAPAN) was used to capture images at 0 and 24 h to assess the extent of cell migration.

### Cell invasion assay

An invasion assay was carried out using a pair of modified chamber plates with a pore size of 8 μm. Matrigel (BD Biosciences, NJ, USA) coated inserts (8 μm pore size, Corning, NY, USA) were seeded with 769-P and ACHN at 5 × 10^4^ cells/well in a serum-free culture medium in the up-committed chamber. Non-invaded cells were gently scraped off the tip of the Matrigel after 24 h of incubation at 37 °C using a cotton-tipped brush. At the bottom of the Matrigel, invaded cells were fixed in methanol, stained with crystal violet, and counted.

### Xenograft murine model and immunohistochemical assays

For the xenograft tumor growth assay, nude mice (Hunan SJA Laboratory Animal Co., Ltd.) were divided into two randomised groups of six mice each and 3 × 10^6^ cells stably transfected with CBX3 knockdown (CBX3-KD) or vector in 0.1 mL volume were injected subcutaneously to establish the ccRCC xenotransplantation model. Tumor growth was monitored and measured in volume using calipers (shorter diameter^2^ × longer diameter/2) every second day. At the end of three weeks, the mice were euthanised, and the tumors were removed for weight measurement. Tumor sections were stained for E-cadherin (ECAD), vimentin (VIM), and CBX3 using 4% paraformaldehyde mixture and paraffin-embedded material, following the supplier’s guidelines. A Zeiss microscope (Oberkochen, Germany) was used for imaging. The whole experiment was carried out at the Shantou University Medical College Animal Experimental Centre, having obtained ethical approval from the Shantou University Medical College Ethics Committee (SYXK2017-0079).

### Differentially expressed genes and functional enrichment analysis

Subjects were stratified based on their CBX3 expression levels using the median cut-off value. Pooled samples were divided into groups with high- and low- CBX3 expression levels. Using the criteria of |log2FC| > 0 and an adjusted p-value (padj) of < 0.05, DEGs were screened. The potential biological functions and mechanisms of action of CBX3 were also explored. Kyoto Encyclopedia of Genes and Genomes (KEGG) and GeneOntology (GO) enrichment results were considered significant at P < 0.05. Screening criteria for Gene Set Enrichment Analysis (GSEA) required a normalized enrichment score |NES| > 1, padj < 0.25 and p-value < 0.05. The GSEA of GO and KEGG were performed individually on the sequenced gene set.

### Correlation analysis of the relationship between CBX3 and PI3K/AKT pathway-enriched genes

The association of CBX3 with PI3K/AKT pathway-enriched genes was also analyzed. Genes associated with CBX3 were determined using a correlation coefficient cut-off of |0.4| and p-value < 0.05.

### Western blotting assay

Protein from the cell lines was completely isolated using a whole-cell protein extraction kit (Beyotime Biotechnology, Jiangsu, China) for westernblotting analysis. Protein samples were resolved on 10% sodium dodecyl polyacrylamide gels (SDS-PAGE) and the protein complexes were transferred to polyvinylidene fluoride blocking membranes (Millipore, USA) with a blocking buffer (Beyotime Biotechnology, Jiangsu, China) for 1 h at 20 ℃. In the presence of primary antibodies, the membranes were then incubated overnight at 4 ℃. After membrane washing, incubation with the following secondary antibody (Proteintech, China) was performed at 20 ℃ for 1 h. The protein bands were seen by enhanced chemiluminescence (Bio-Rad, Hercules, CA, USA). Proteintech (Jiangsu, China) provided primary antibodies against CBX3, Bax, Bcl-2, VIM, NCAD, GAPDH, and E-cadherin (ECAD). Cleaved polymerase (ADP-ribose) (PARP), AKT, PI3K, and phosphorylated (p)-AKT antibodies were obtained from Cell Signalling Technology (Danvers, MA, USA).

### Exploration of the relationship between CBX3 and immunity in KIRC

The immune scores of the Step1-Step7 cancer immunity cycle from the tracking of the tumor immunophenotype (TIP) [20] were employed for all samples to assess the degree of the immune response and comparethe KIRC tumor and normal samples. The same procedure was applied to samples with high or low CBX3 expression. We also observed that the cancer immunity steps were correlated with survival outcomes in patients KIRC. Furthermore, the correlation between the accumulation of 24 immunocyte types and the level of CBX3 expression was investigated using the online tool ‘ImmuCellAI’ [20]. We collected KIRC immunological subtype data from the Tumor Immune System Interaction Database (http://cis.hku.hk/TISIDB/) [22]  to examine their relationship with prognosis. The CancerSEA [23] database was used to illustrate the relationship between CBX3 expression and renal cancer function.

### Analysis of potential chemotherapy drugs

To predict CBX3 sensitivity to immune checkpoint inhibitors (ICIs), the correlation of CBX3 with immune checkpoints was analyzed using TCGA-KIRC datasets. To further determine the relationship between CBX3 levels and drug susceptibility, data, including the z-score of NCI-60 medication activity and information on the expression of genes in cancer cell lines, were obtained from the CellMiner database [23].

### Statistical analysis of data

Each experiment was conducted with a minimum of three replicates. The resulting data are presented as the mean ± standard deviation. The statistical studies were performed using the SPSS program (v17.0; SPSS Inc., USA). Any statistically significant findings related to the experiments were evaluated using the Student’s *t*-test. The R software (v 4.2.2) was used for data visualisation and statistical analyses. Relationships between continuous variables were examined using Pearson's/Spearman's correlation analyses. For continuous variables, two or three treatment groups were compared using Kruskal–Wallis and Wilcoxon rank-sum tests, respectively. P-values < 0.05 or padj < 0.05 were deemed statistically significant.

## Results

### CBX3 expression levels increased in the KIRC and ccRCC cell lines

**Fig. 1:**
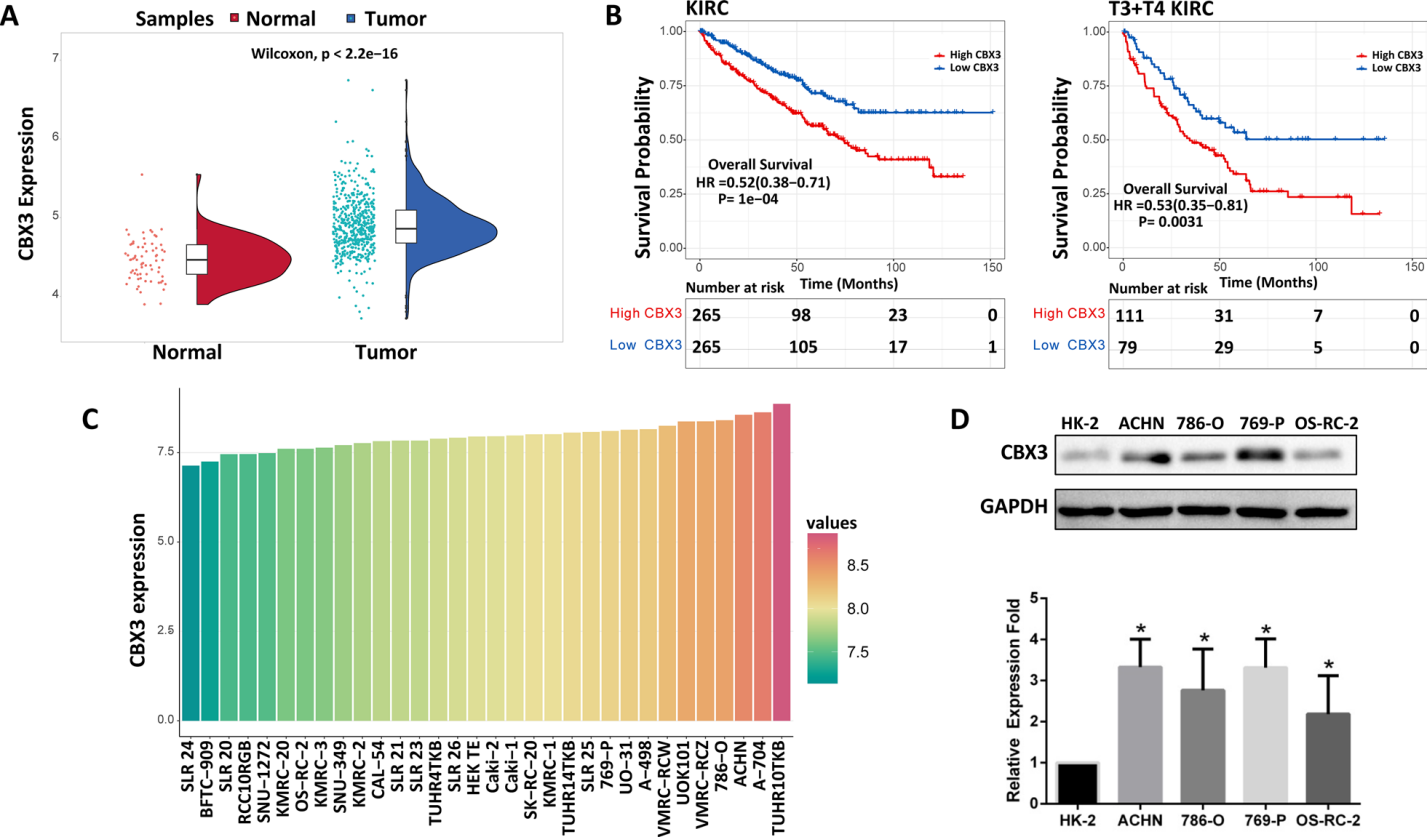
CBX3 expression and survival status in TCGA-KIRC databases: **A** CBX3 expression in KIRC tissues compared to that in normal tissues. **B** Survival status linked to CBX3 expression in patients with KIRC, especially in T3 and T4 stages. **C** CBX3 expression distribution in kidney cancer cell lines. The ordinate represents the expression distribution of CBX3 and the abscissa represents different cell lines and different colors and the height of the bots represent CBX3 expression levels. **D** Western blotting assays for detecting CBX3 expression in ccRCC cells. *P < 0.05 is statistically significant

To thoroughly analyze the presence of CBX3 in KIRC samples, we initially studied the changes in CBX3 expression levels between KIRC and matched normal tissues and found that expression of CBX3 was markedly increased in tumoral tissues (Fig. 1A). We also collected and analyzed gene expression data from 33 kidney cancer cell lines and observed that CBX3 was expressed similarly across all cell lines (Fig. 1C). In addition, a western blotting assay was conducted to assess CBX3 expression levels in ccRCC cell populations and the normal kidney cell line HK-2. The cell lines 769-P and ACHN, which expressed the highest level of CBX3 (Fig. 1D), were selected for subsequent analyses. We looked at the correlation between CBX3 transcription and clinical outcomes based on the pathologic stages T of tumors in order to comprehend the function of CBX3 in the development of KIRC (Fig. 1B). Prognostic results related to CBX3 in other pathological stages are shown in Additional file 2: Fig. S2. The findings demonstrated a substantial relationship between CBX3 overexpression and poor overall survival (OS), particularly in individuals with T3 and T4 illness. These results imply that CBX3 participated in the carcinogenesis and development of KIRC.

### Knockdown of CBX3 expression inhibited proliferation and induced apoptosis of renal cancer cells

**Fig. 2:**
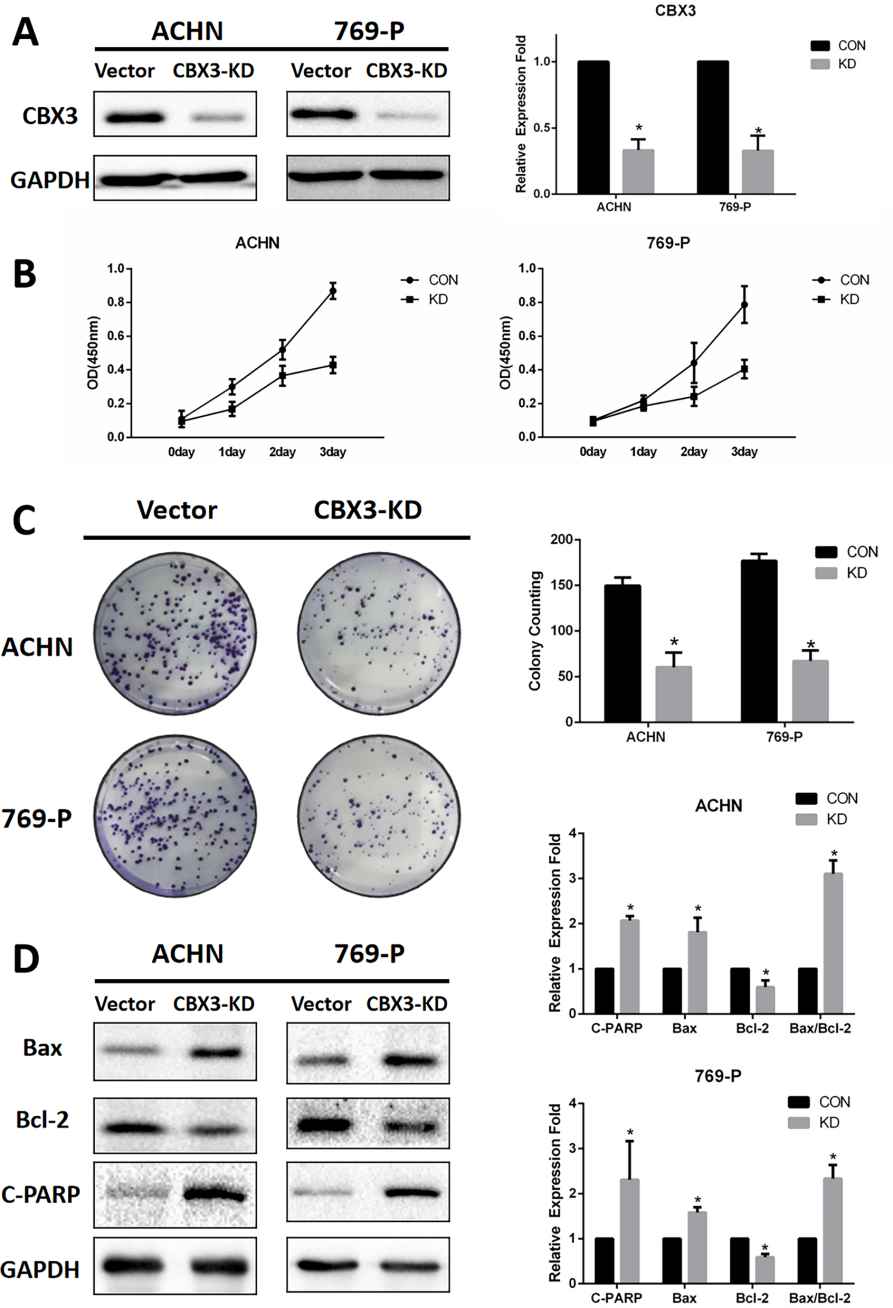
Knockdown of CBX3 in ccRCC cells inhibited cell growth and induced cell apoptosis. **A** The expression levels of CBX3 were examined via western blotting. **B** A Cell Counting Kit-8 assay was used to detect the levels of cell proliferation. **C** A colony formation assay was used to detect cell cloning levels. **D** Western blotting was used to detected the expression levels of apoptosis-related proteins (Bax, Bcl-2 and cleaved PARP). *P < 0.05 is statistically significant

CCK-8 and plate colony formation assays were employed to analyze the involvement of CBX3 in ccRCC cell expansion, in order to assess the impact of CBX3-KD on ccRCC cells multiplying. CBX3-KD group exhibited inhibited cell viability and reduced the quantity and size of colonies compared to the control group (Fig. 2B, C). Furthermore, western blotting examination revealed that the amount of cleaved PARP, an apoptosis marker, increased considerably in the CBX3-KD group compared to the controls. The expression of Bcl-2, an anti-apoptotic factor, reduced, whereas that of Bax, a pro-apoptotic factor, increased (Fig. 2D). These findings suggest that CBX3 plays a crucial role in RCC cell proliferation and apoptosis.

### Knockdown of CBX3 expression inhibited emigration, invasion, and epithelial-to-mesenchymal transition of renal malignancy cells

**Fig. 3:**
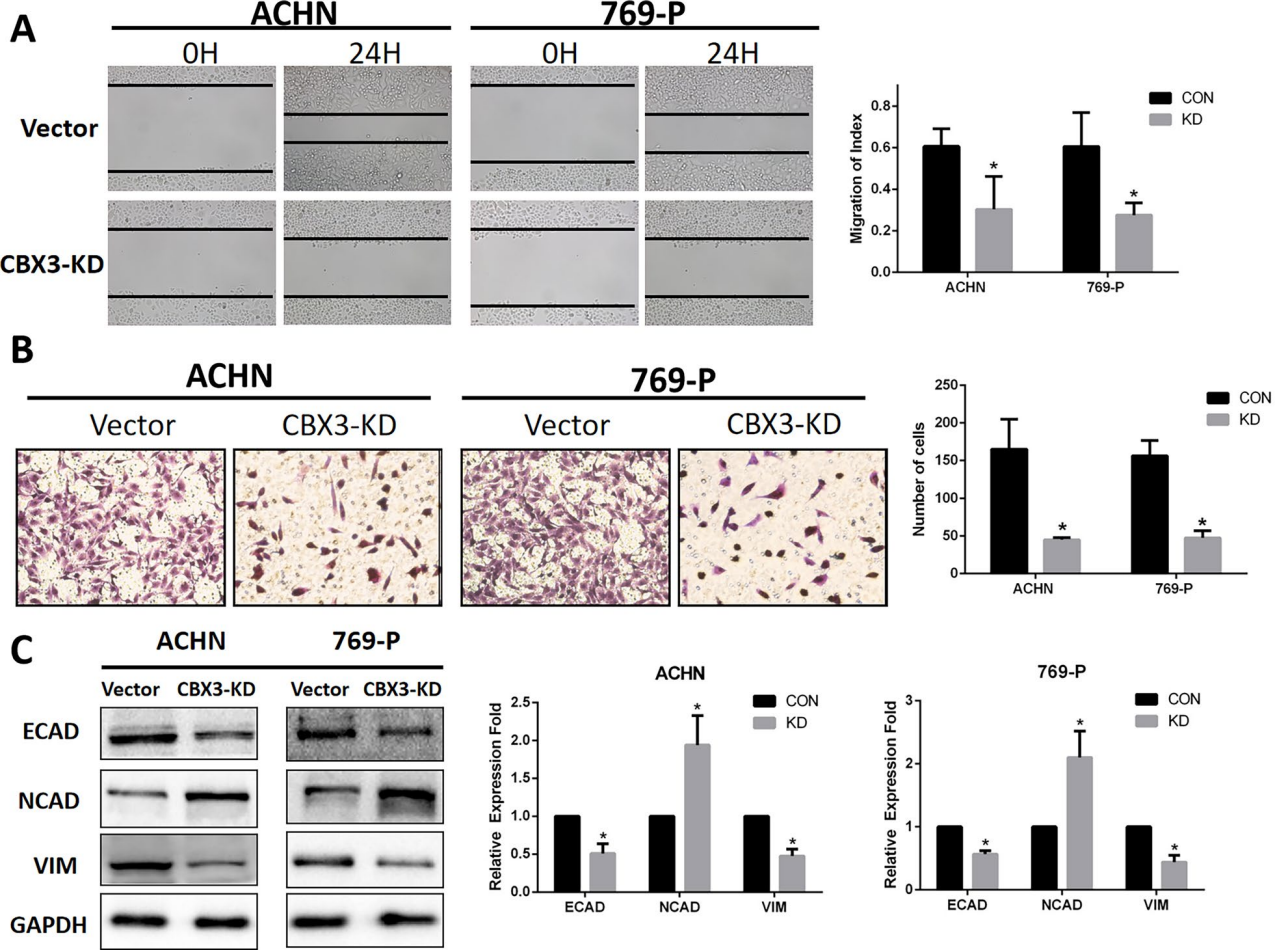
Knockdown of CBX3 inhibited cell migration, inversion and EMT process in ccRCC cells in vitro. **A** Wound-healing and **B** Transwell assays were used to detect the levels of migration and invasion of ccRCC cells. Scale bar, 100 µm. Expression levels of **C** EMT-related proteins (ECAD, NCAD, VIM) were detected via western blotting. *P < 0.05 is statistically significant

To validate the effect of CBX3-KD on the migration of renal cancer cells, we performed wound healing assays. The results indicated reduced wound closure in ccRCC cells in the CBX3-KD group compared to those in the control group (Fig. 3A). In the transwell cell invasion assays, the results showed that CBX3-KD dramatically decreased the invasive ability of ccRCC cells (Fig. 3B). EMT is an important pathway that promotes renal carcinoma migration and metastasis. To better understand the effects of CBX-KD on RCC cells, we analyzed the generation of EMT process-associated molecules (ECAD, VIM, and NCAD). The results showed that CBX3-KD dramatically increased the levels of NCAD protein while decreasing the production of ECAD and VIM proteins in ACHN and 769-P cells (Fig. 3C). These data suggest that CBX3 expression altered ccRCC EMT, thus influencing the migration and invasive capacities of tumor cells.

### CBX3 knockdown inhibited tumor progression in vivo

**Fig. 4:**
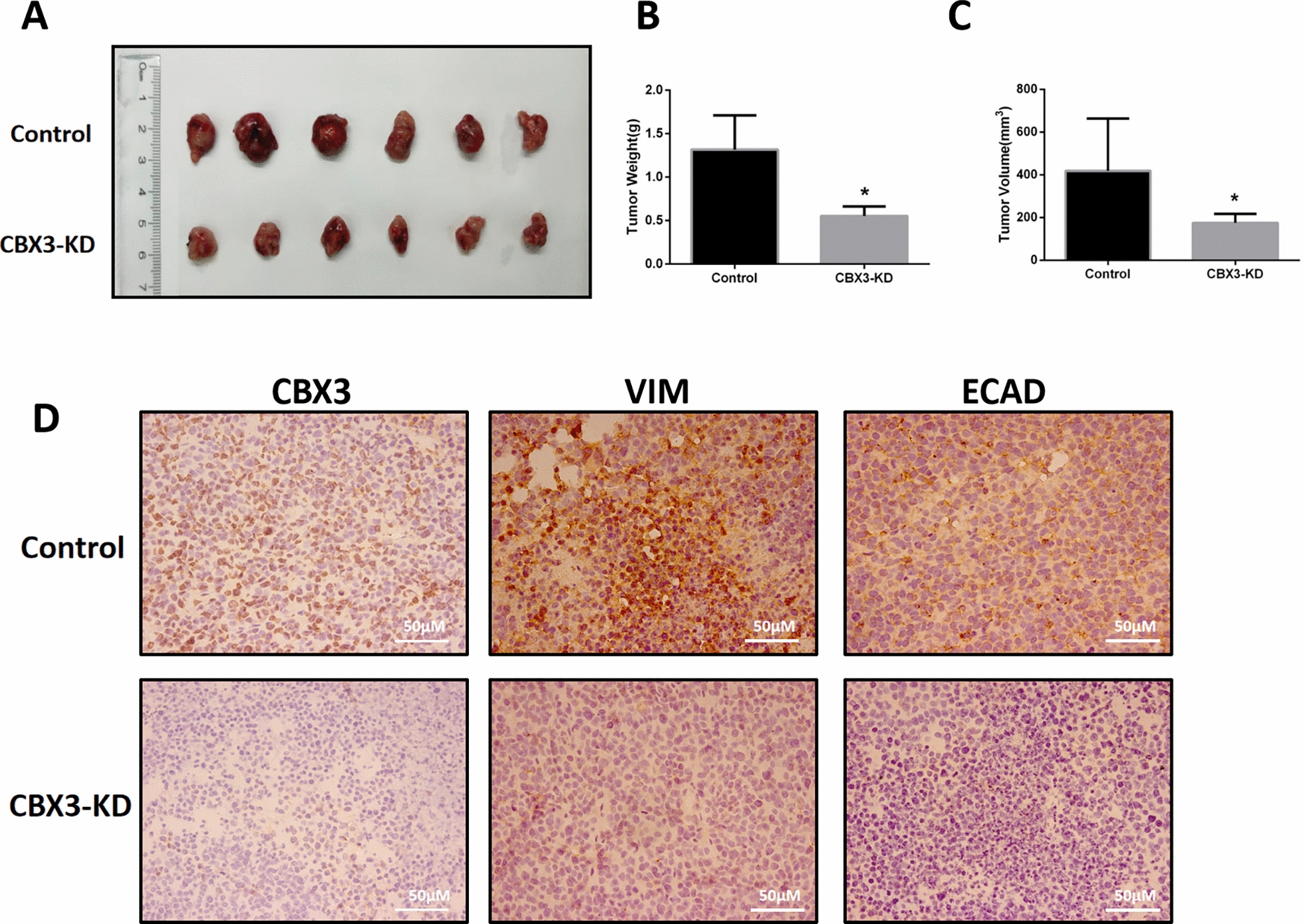
Knockdown of CBX3 inhibited ccRCC cells growth in vivo. **A** CBX3-KD significantly inhibited tomor growth in nude mice xenograft. The tumor volume **B** and weights **C** are shown in the right panel. **D** The expression of Ki-67, ECAD and VIM in tumor tissues were analyzed by immunohistochemistry (IHC) staining (400 ×)

A 769-P xenograft model was created to study the involvement of CBX3 in ccRCC in vivo. The volume of the tumor was measured every 3 d and mice were euthanized 4 weeks after inoculation to determine tumor weight. Compared to the control group, the CBX3-KD group exhibited suppressed tumor progression, as evidenced by considerably reduced tumor size and weights (Fig. 4A–C). Furthermore, the immunohistochemistry test revealed that the CBX3-KD group exhibited lower expression levels of CBX3 and the EMT-related genes (ECAD, and VIM） (Fig. 4D). Notably, these results were also confirmed in the previous in vitro assay.

### CBX3 activated the signaling pathway mediated by PI3K/AKT in ccRCC

The genes co-expressed with CBX3 and their associated enrichment functionswere investigated to identify the underlying mechanisms by which CBX3 promotes ccRCC development. In total, 17,279 DEGs were identified in both the low- and high- CBX3 expression groups in accordance with the screening conditionsThe level of expression and distribution of these DEGs, including 11,484 upregulated DEGs and 5795 downregulated DEGs in KIRC, are shown in a volcano plot (Additional file 1: Fig. S1A). A radar plot (Additional file 1: Fig. S1B) was developed to highlight the top 15 upregulated DEGs and the top 15 downregulated DEGs.

**Fig. 5:**
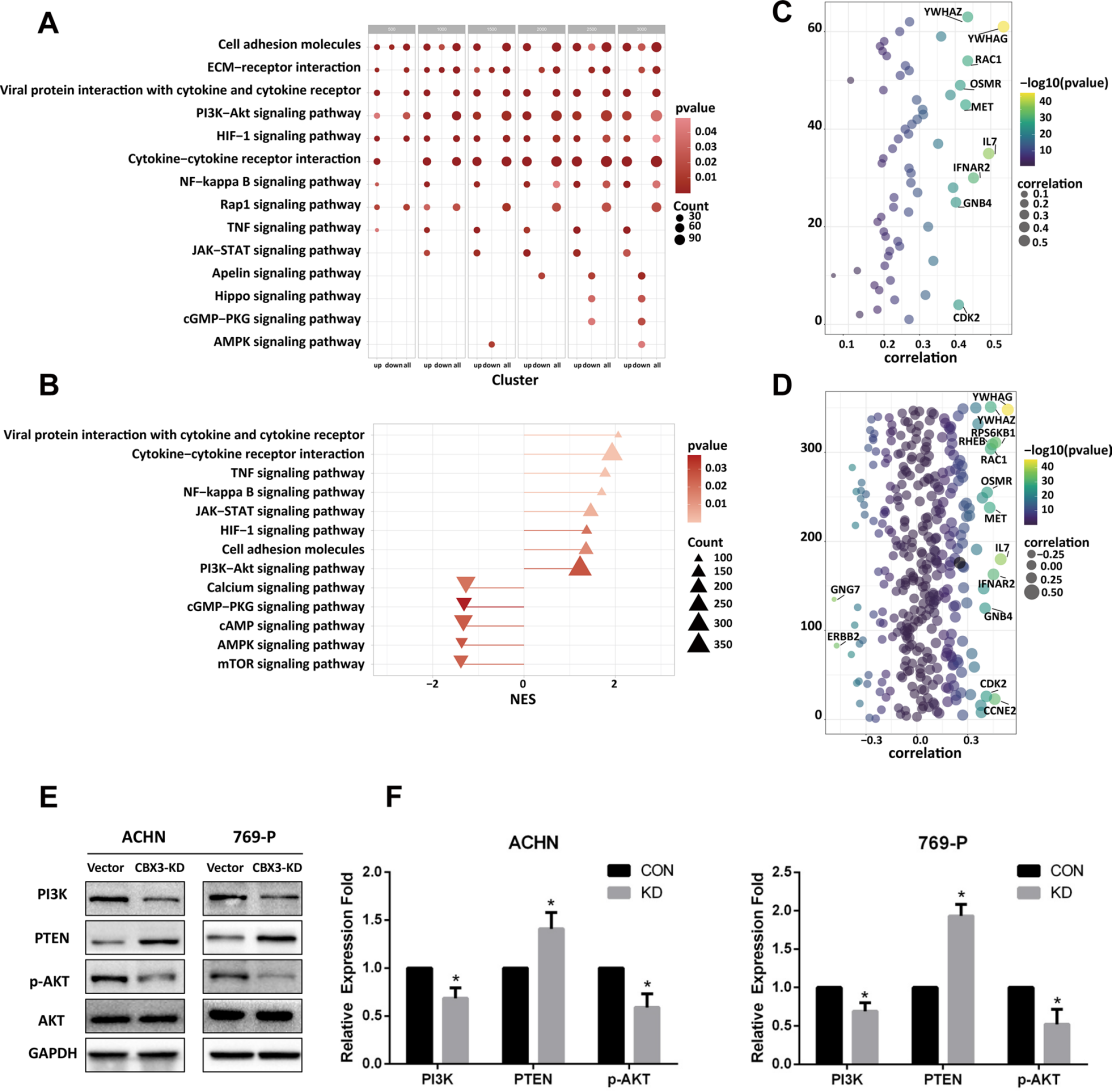
Exploration of a relationship between CBX3 and PI3K/AKT signaling pathway. **A** KEGG functional enrichment analysis of selected genes in bubble chart (p-value < 0.05). **B** Prominently enriched pathways as determined by GSEA (P-value < 0.05, padj < 0.25 and |NES|> 1). Correlation between CBX3 and enriched genes in PI3K/AKT signaling pathway by KEGG functional enrichment analysis (**C**) and GSEA-based KEGG analysis (**D**). The genes were significantly associated with CBX3 by the correlation coefficient which exceeded |0.4|. The details in correlation between CBX3 and significantly enriched genes in PI3K/AKT signaling pathway see Additional file 3: Fig. S3. **E**, **F** Western blotting was used to detect the expression levels of PI3K/AKT pathway related proteins. *P < 0.05 is statistically significant

To explore the mechanisms by which CBX3 regulates migration and invasion, 500 upregulated and downregulated genes and all DEGs underwent progressive KEGG enrichment analysis. Interestingly, upregulated DEGs were consistently enriched in the ‘PI3K/AKT signaling pathway’, ‘Viral protein interaction with cytokine and cytokine receptor’, and ‘HIF-1 signaling pathway’, regardless of the increment in gene count (Fig. 5A). To further investigate the probable molecular pathways underlying the impact of CBX3, we performed a GSEA-based KEGG analysis. Thirteen important KEGG pathways, comprising both stimulated and inhibited processes, were identified (Fig. 5B). The activated pathways mainly included the above-mentioned pathways, whereas the suppressed pathways mainly comprised the AMPK and mTOR signaling pathways. GSEA outcomes further validated the results of the KEGG functional enrichment analyses, accentuating a strong correlation between CBX3 and the PI3K/AKT signaling pathways. To better elucidate this link, we conducted a correlation study, analysing the relationship between between CBX3 activity and genes in the PI3K/AKT signaling pathway (Fig. 5C, D). Of these, YWHAG, YWHAZ, RAC1, OSMR, MET, IL7, IFNAR2, and GNB4 were highly associated with CBX3 expression.

Additionally, western blotting analysis was performed on RCC cells to evaluate components of the PI3K/AKT pathway. Our data showed that CBX3-KD expression lowered the amounts of phosphorylated (active) forms of AKT and PI3K in ACHN and 769-P cells, but increased PTEN expression (Fig. 5E, F). These results also confirmed our bioinformatics analysis, establishing the role of CBX3 in activating the PI3K/AKT pathway in ccRCC. These findings imply that CBX3 takes part in the activation of the PI3K/AKT pathway in ccRCC.

### CBX3 influenced immunity in KIRC

**Fig. 6:**
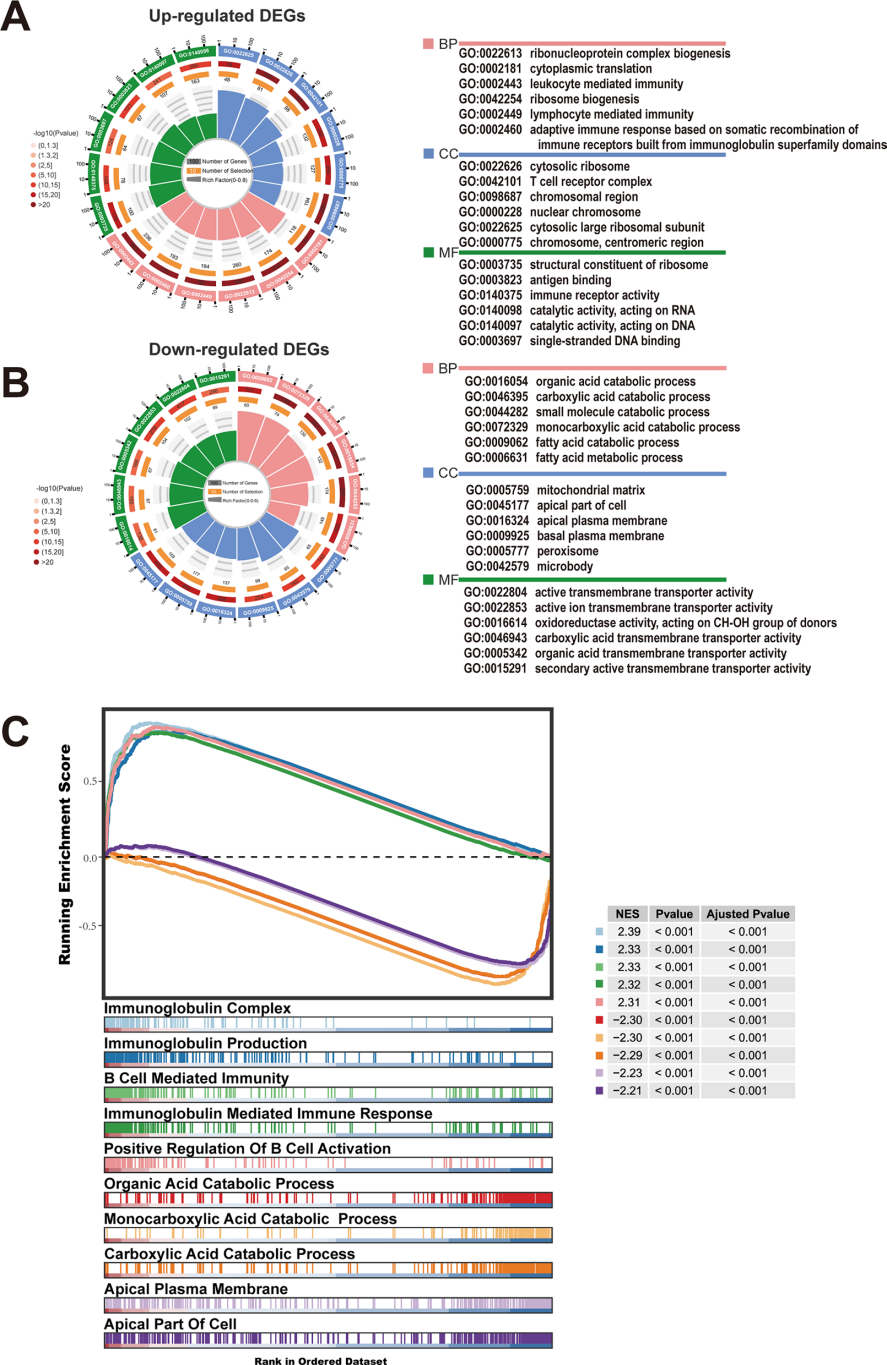
The enrichment results of GO based on functional enrichment analysis and GSEA analysis. GO functional enrichment analysis of up-regulated DEGs (**A**) and down-regulated DEGs (**B**) in loop graph (P < 0.05). **C** Prominently enriched GO terms as determined by GSEA (P < 0.05, padj < 0.25 and |NES|> 1)

To determine the biological characteristics of CBX3,  a functional enrichment analysis of GO was performed. The GO analysis findings (Fig. 6A) revealed that the elevated genes were predominantly connected with immune-related terms such as ‘immune receptor activity’ in molecular function, ‘T cell receptor complex’ in cellular component and ‘lymphocyte-mediated immunity’ in biological process. However, the analysis of downregulated genes revealed that these genes were linked to transmembrane carrier activity and acid catabolic processes, such as ‘active ion transmembrane transporter activity’ and ‘fatty acid catabolic process’ (Fig. 6B). We performed a GSEA-based GO analysis to further investigate the probable molecular processes affected by CBX3. Figure 6C shows the top five significantly enriched GO terms according to |NES| for both upregulated and downregulated genes. The top five terms upregulated were mostly related to the immunological activity of B cells, such as ‘immunoglobulin mediated immune response’ and ‘B Cell-mediated Immunity’. However, the top five downregulated genes were concentrated in the cellular elements of the apical and associated acid catabolic processes. The results of the GO functional enrichment analysis and GSEA agreed, suggesting that CBX3 positively controls immunological function and negatively controls acid catabolic processes in KIRC.

### Immunity disorders caused by CBX3 in KIRC

**Fig. 7:**
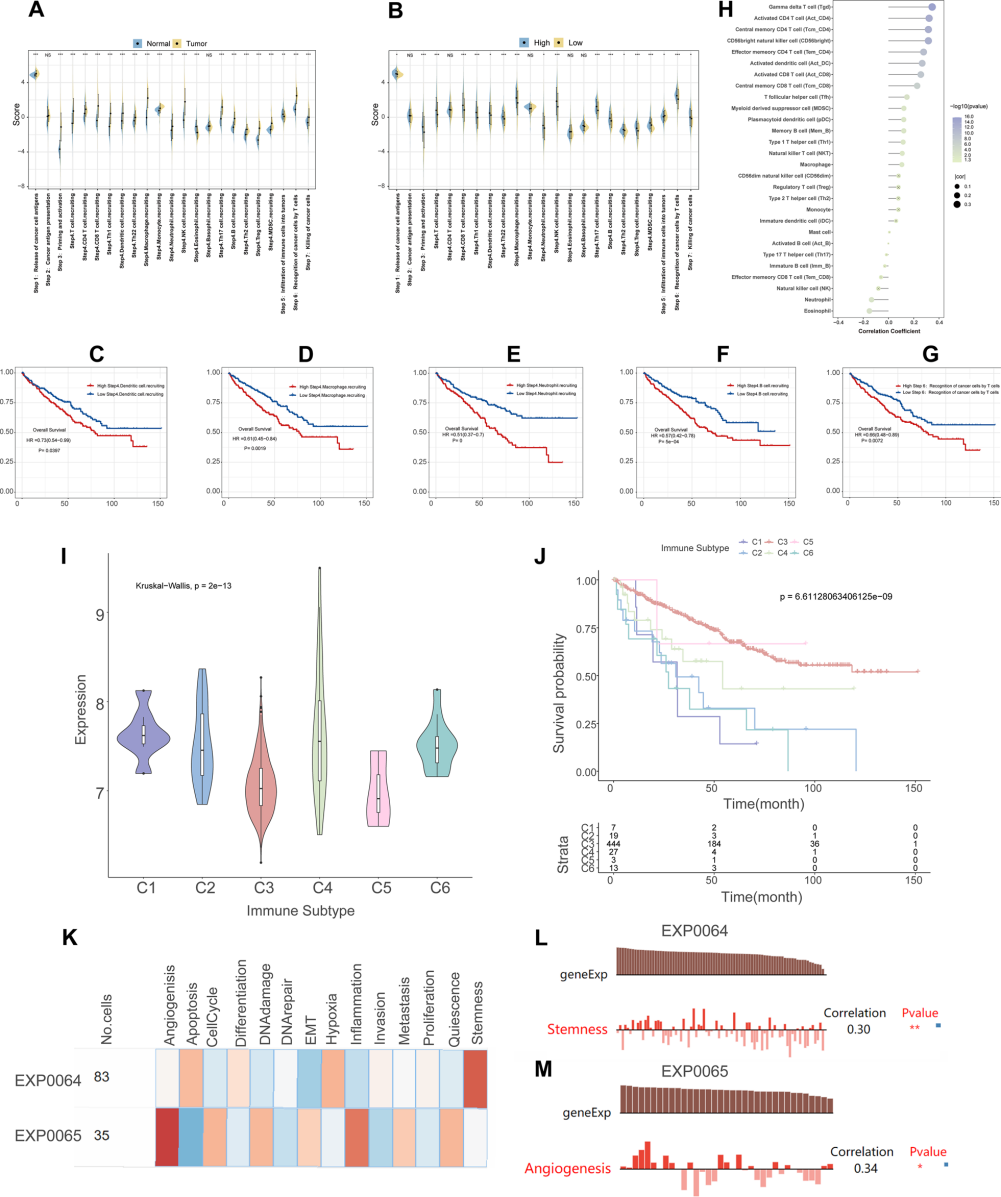
The effects of CBX3 on immunity in KIRC. **A** The differences in the activity levels of the seven steps in the cancer immunity cycle between the normal and tumor groups. **B** The differences in the activity levels of the seven steps in the cancer immunity cycle between high and low CBX3 expression groups. Prognostic predictive power of the cancer immunity steps, including step 4 for recruitment of dendritic cells (**C**) and macrophages (**D**), neutrophils (**E**) and B cells (**F**), and step 6 for recognition of cancer cells by T cells (**G**). **H** The correlation between the level of 24 infiltrating immune cells and CBX3 expression. **I** Relationship between CBX3 and immune subtypes in KIRC. **J** Effects of immune subtypes on survival of KIRC patients. **K**–**M** The association between CBX3 expression and kidney cancer function in EXP0064 and EXP0065. C1: wound healing (n = 7); C2: IFN-gamma dominant (n = 20); C3: inflammatory (n = 445); C4: lymphocyte depleted (n = 27); C5: immunologically quiet (n = 3); C6: TGF-β dominant (n = 13). Significance levels:*p < 0.05; **p < 0.01; ***p < 0.001

The GO enrichment results, which indicated that CBX3 was strongly associated with immunity, encouraged us to further investigate the role of CBX3 in the immunological system in KIRC. Immunity cycle analysis (Fig. 7A, B) showed that several processes, including step-1 (release of cancer cell antigens), step-3 (priming and activation), step-4 (recruiting CD8^+^ T cells, T cells, Th1 cells, Th2 cells, Th17 cells, Th22 cells, B cells, Tregs, NK cells, dendritic cells (DCs), neutrophils, macrophages and myeloid-derived suppressor cells), step-6 (recognition of cancer cells), and step-7 (killing of cancer cells), were significantly more active in the KIRC group with high CBX3 expression. In contrast, KIRC and high expression of CBX3 were linked to reduced step-5 activity (infiltration of immune cells into malignancies). Next, we proceeded with prognostic predictions centred on immune steps to further investigate the prognostic relevance of various immune-responsive processes in KIRC. Figure 7C–F shows a link between an activated immune process that recruits DCs, macrophages, neutrophils, and B cells and a lower OS. And the poor prognosis of KIRC was associated with an elevated active level of the process of detecting cancer cells by T cells (Fig. 7G). We also examined the potential involvement of CBX3 in the invasion of immune cells. Figure 7H shows that CBX3 expression had a significantly positive relationship with macrophages, memory B cells, and plasmacytoid dendritic cells (pDCs) in KIRC, whereas CBX3 expression was negatively associated with neutrophil infiltration. The change in the proportion of immune-infiltrating cells associated with CBX3 in KIRC is shown in Additional file 5: Fig. S5. Finally, we explored how the immunological subtypes of KIRC were affected by CBX3 expression. CBX3 expression varied among immune subtypes and that the prognosis of KIRC patients also varied based on immune subtypes (Fig. 7I, J). Furthermore, angiogenesis and stemness were positively correlated with CBX3 expression in kidney cancer (Fig. 7K–M). In summary, the above findings indicate that CBX3 potentially promoted cancer development by affecting immunity in KIRC.

### Exploration of drugs that potentially inhibit KIRC development

**Fig. 8:**
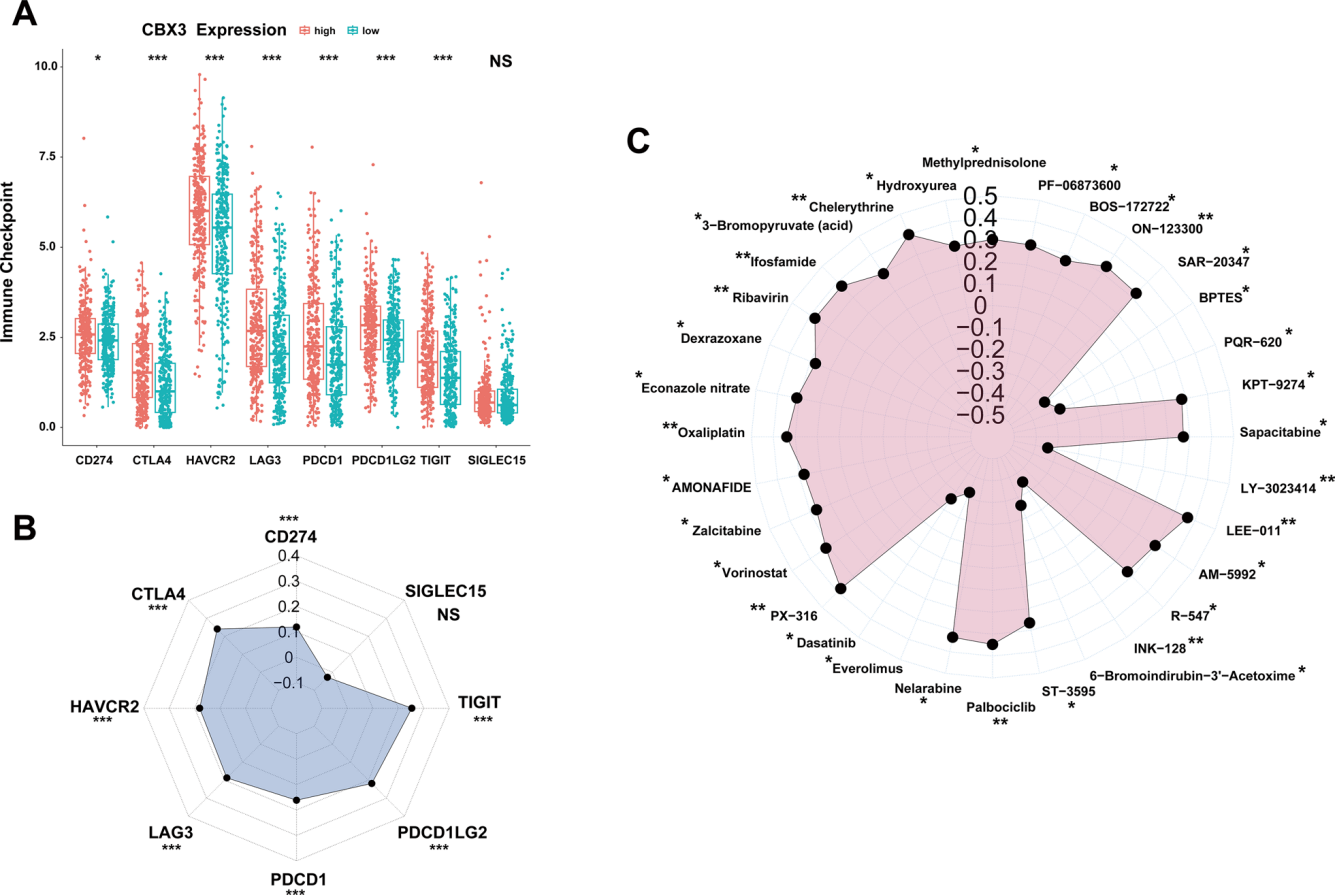
Potential drug sensitivity assessment of CBX3 expression. **A** Variations in the expression of immune-checkpoint-related genes between CBX3 high- and low-expression groups. **B** Correlation analysis on expression between immune checkpoints and CBX3. **C** Correlation analysis of CBX3 expression with activity score of different drugs acquired from the CellMiner database. *p < 0.05, **p < 0.01, ***p < 0.001

To provide KIRC patients with more effective treatment options, we investigated potential chemotherapeutic drugs that inhibit the CBX3-regulated tumorigenic process. LAG3, CD274, CTLA4, TIGIT, PDCD1, HAVCR2, SIGLEC15, and PDCD1LG2 were selected as immune checkpoints. All genes, except for SIGLEC15, were more abundant in the CBX3 high-expression group and had a positive association with CBX3 expression (Fig. 8A, B). These findings suggested that KIRC patients with high expression of CBX3 would respond well to ICIs. Thirty-two significantly associated drugs were obtained from the CellMiner database. CBX3 expression was negatively related to INK-128, LY-3023414, everolimus, BPTES, PQR-620, 6-bromoindirubin-3′-acetoxime, and dasatinib but was positively related to the remaining genes (Fig. 8C). In summary, patients with high-CBX3 expression may respond favourably to treatment with some ICIs, along with the 25 drugs positively related to CBX3.

## Discussion

Recently, targeted drug and immune checkpoint inhibitor therapies have become very important for metastatic ccRCC; however, patients still develop drug resistance after several months [25, 26]. Therefore, it is necessary to identify the key genes associated with ccRCC to determine potential treatment targets.

Growing evidence indicates that CBX3 is crucial for the incidence, progression, and prognosis of numerous kinds of cancers, including ccRCC. Previous research has linked elevated CBX3 expression to poor outcomes in lung, gastric, and ovarian malignancies [27–29]; however, a study has also shown that CBX3 expression can have a tumor-suppressive effect in malignant glioma [30]. These studies suggest that CBX3 functions as a promotor and suppressor in particular cancer types. However, research on the link between CBX3 and ccRCC is minimal. Previous research has demonstrated that CBX3 is an oncogene associated with poor outcomes in ccRCC; however, the function and potential mechanism of CBX3 remain unclear [31]. Therefore, we investigated the role of CBX3 in ccRCC.

Initially, we discovered that CBX3 was substantially expressed in renal carcinoma, concerning a dismal prognosis—especially in high-grade (T3 + T4) ccRCC; these findings agreed with those of earlier research [31]. The analysis of the CCLE datasets revealed that CBX3 was substantially expressed in kidney cancer cell lines. The western blotting confirmed that CBX3 was significantly upregulated in ccRCC cells. These results indicate that an increase in CBX3 expression can result in tumorigenicity in ccRCC, thus supporting the view that CBX3 may function as a potential oncogene, which can encourage the development and metastasis of ccRCC. To verify our hypothesis, we investigated the effects of CBX3-KD. CBX3-KD decreased ccRCC cell growth, migration, and invasion in vitro, complemented by reduced ccRCC cell tumorigenicity in vivo. Additionally, western blotting showed that CBX3-KD rendered cells more susceptible to apoptosis.

Mechanisms underlying EMT have gained importance in explaining the invasive progression and metastasis of ccRCC. A defining feature of EMT is the degradation of ECAD, which reinforces the instability of adherent connections, the increase in the mesenchymal signature NCAD, and an upsurge in the protein density of VIM. Our bioinformatics analysis revealed that CBX3 was significantly correlated with ECAD, NCAD, and VIM expression, and western blotting studies displayed that the suppression of CBX3 expression dramatically decreased the expression of ECAD, NCAD, and VIM in 769-P and ACHN cell lines. Taken together, our findings imply that CBX3 bolsters the metastasis of ccRCC cells, which may be correlated with the process of EMT.

To clarify the mechanism underlying CBX3 effects in ccRCC, we performed a bioinformatic analysis of CBX3 based on the TCGA dataset. Our data indicated that CBX3 and its associated genes (upregulated or downregulated) were involved in several pathways, including the PI3K/AKT signaling pathway. The PI3K/AKT pathway has been implicated in tumor cell metastasis and invasion, and regulates EMT in various tumors, including ccRCC. This pathway activates VIM transcription by upregulating PI3K (phosphatidylinositol kinase) to phosphorylate and activate Akt [32, 33]. In the present study, CBX3-KD decreased PI3K and p-AKT expression but increased PTEN expression (a PI3K/AKT pathway suppressor). Thus, we further investigated the relationship between PI3K-related genes and CBX3 (Additional file 6: Fig. S6). We found that PIK3R6, which encodes the regulatory subunit of the PI3K gamma complex [34], was significantly upregulated in the high-CBX3 expression KIRC group, regardless of metastasis status, and was more pertinent to CBX3 in the metastasis samples (Additional file 7: Fig. S7A1, A2, B1, B2). Along with CBX3, PIK3R6 was associated with poorer prognosis in KIRC (Additional file 7: Fig. S7C), which agrees with the findings of a previous study [35]. These observations suggest that the PI3K/AKT pathway underlies the functional role of CBX3. As CBX3 may act as an upstream regulator of the PI3K/AKT pathway in ccRCC, it is essential to evaluate the mechanisms of action of CBX3 to assess its potential as a drug target.

The incidence and course of KIRC, a highly immunogenic malignant neoplasm, are closely correlated with TME [36, 37]. According to the results of our GO analysis, most immune-related elements were the target of upregulated DEGs, indicating that CBX3 is likely implicated in immunomodulatory disorders in patients with KIRC. Our analysis (based on different phases of the immune responses and the relation between immune cell infiltration and CBX3) also suggested that the recruitment of immune cells (i.e., macrophages, pDCs, and B cells) by CBX3 is critical for the development of KIRC. Several pro-inflammatory and pro-angiogenic substances secreted by tumor-associated macrophages, a mixed M1/M2 phenotype that predicts poor prognosis in KIRC [38, 39], promote neovascularization [40, 41], tumor cell hyperplasia [40, 41], and immunosuppression [44]. There is also evidence that pDCs, a subclass of DCs, exhibit a diminished response to TLR7/9 activation and generate less IFN when exposed to the TME [45]. Furthermore, pDCs produce IL-10, which facilitates tumor immune escape by reducing the T-cell response and inducing regulatory T-cell hyperplasia [45]^.^ Recent research has revealed that the presence of increased mature and memory B cell populations in the TME is associated with better clinical outcomes [49–51]. In contrast, there is a negative correlation between B-cell infiltration and OS in KIRC [52]. Saiyang et al. [53] investigated tumor-educated B cells (TEBs) in KIRC and found that more TEBs were recruited in tumor tissues, which activated IL-1 to increase KIRC’s capacity for invasion and metastasis by triggering the PI3K/AKT pathway. This observation is consistent with our findings that CBX3 and PI3K/AKT are closely related. Considering these results, it is plausible to speculate that CBX3 can promote the development of KIRC by recruiting macrophages, pDCs, and B cells during specific phases of immunological derangement.

A correlation between CBX3 expression and the immunological subtypes in KIRC was initially demonstrated in our study. We aimed to elucidate the mechanisms underlying CBX3’s influence on immunity in KIRC through comprehensive analyses of our results and insights from existing literature. The highest level of CBX3 expression was observed in the C1 subtype (wound healing), and patients with this subtype had the poorest prognoses. A previous study [54] demonstrated that the C1 subtype promotes angiogenesis and cell proliferation. A positive correlation between CBX3 and angiogenesis and stemness in KIRC was discovered through the cancerSEA database. It was universally known that the PI3K pathway can promote angiogenesis and tumor cell proliferation, consistent with the results of our immunosubtype analysis. Patients with subtype C3 (inflammatory), the primary subtype in normal kidney tissues had the best prognoses[54]. Reduced CBX3 expression was observed in the C3 subtype, indicating that CBX3 may be crucial in reversing the normal immune response of the kidney. Interestingly, CD40 amplification and gene mutation drivers, such as TP53, PIK3CA, and PTEN, were particularly prevalent in the C1 and C2 (*IFN*-gamma dominant) subtypes [54]. CD40, a costimulatory receptor in B cells and a member of the TNF receptor family, influences the fate of B cells [55]. Previous studies have shown that CD40 signaling can stimulate B cell expansion and differentiation and regulate the development of various memory B cell subtypes [56–58]. Furthermore, aberrations in these driver genes have been widely demonstrated to cause hyperactivation of the PI3K signaling pathway. This pathway plays pivotal role in the growth and activation of B-lymphocytes, and the proliferation of cancerous B cells [58]. Moreover, memory B cells activate PI3K signaling [53], consistent with our GSEA-GO results, which found that CBX3 was highly linked to the B cell immune response. Furthermore, a previous study established an association between tumor stemness and CBX3 levels, and its overexpression was associated with higher mutational loads in pancreatic adenocarcinoma, lung adenocarcinoma, hepatocellular carcinoma, and endometrial cancer [53]. The cancerSEA database investigation revealed a positive correlation between CBX3 expression and tumor stemness in kidney cancer. Therefore, it is plausible to suggest that CBX3 initiates gene modifications that activate the PI3K signaling pathway, leading to immunological disorders and tumor growth in KIRC. More importantly, CBX3 may amplify the effects of vascularization and cell proliferation (induced by the PI3K signaling pathway) by regulating immunity.

Our findings on the effects of CBX3 on the TME demonstrate how crucial the PI3K pathway and CBX3 are in mediating immunological disorders in KIRC. Thus, we used immunoassays to study PIK3R6, the PI3K-related gene with the strongest correlation with CBX3. In the CBX3-high expression group, increased in PIK3R6 expression levels were associated with score increases (EstimateScore, StromalScore, ImmuneScore) and decreases in tumor purity (Additional file 7: Fig. S7D). Increased in CBX3 expression levels alone were not associated with changes in these scores, according to a previous study [60] and the results of the present study (Additional file 4: Fig. S4). Thus, CBX3 may trigger immunological disorders by stimulating the PI3K pathway. Additionally, PIK3R6 resembles CBX3 and is more closely associated with the presence of B cells and macrophages than CBX3 (Additional file 7: Fig. S7E). Deficits in the PI3K gamma complex have been linked to migratory abnormalities in DCs [34]. These findings further suggest that CBX3 can influence tumor immune dysfunction and development by enhancing PIK3R6 expression to activate the PI3K pathway.

Based on the immunological investigations in this study, CBX3 appears promising as an immunotherapeutic target for KIRC. We explored potential drug interventions targeting CBX-3 to identify viable treatment options for KIRC and strategies to circumvent drug resistance. First, according to an analysis of the relationship between CBX3 and immunological checkpoints, KIRC individuals with high expression of CBX3 may be more susceptible to ICIs. Second, evaluation of the possible impact of CBX3 on the response to anticancer treatment revealed that cyclin-dependent kinase (CDK) inhibitors dominated the list of agents that were positively related to CBX3 expression. Several studies [61, 62] have shown that various tumor markers may alter the G2/M phase, affecting the regulation of the KIRC cell cycle. This suggests that CBX3 affects cell cycle stages, such as the G2/M phase, of KIRC. Sensitivity to PI3K or mTOR inhibitors, such as LY-3023414, PQR-620, INK-128, and everolimus, was inversely correlated with CBX3 expression, indicating that patients exhibiting high expression of CBX3 may be resistant to these medications. These drugs alter the PI3K-mTOR pathway, promoting differential responses of the pathway to particular therapeutic agents [63, 64]. These results suggest that ICIs and CDK inhibitors that prevent CBX3 -mediated tumorigenesis could serve as potential molecularly targeted therapeutics and that medications that specifically target CBX3 could serve as a strategy to surmount resistance to PI3K/mTOR inhibitors.

While our tests and biometric analyses, as detailed above, provide substantial insights into nearly all of the effects of CBX3 on the PI3K pathway and immunological microenvironment in KIRC, the data generated from our research, although significant, might not encompass the entirety of the effects. Moreover, the biometrical analysis, while informative, lacks direct experimental validation and is instead reliant on several algorithms. Therefore, further research is required to demonstrate the validity of the proposed mechanism.

## Conclusions

In summary, our research reaffirms the adverse prognosis associated with significant CBX3 expression in individuals with ccRCC. Additionally, we demonstrated that CBX3-KD suppressed the migration, invasion, EMT process, and survival of ccRCC cells through the PI3K/AKT pathway. Furthermore, we identified an interdependent relationship among CBX3, tumor immune disorders, and the PI3K pathway. Therefore, our results underscore the potential of CBX3 as a new biomarker for the noninvasive survival and diagnosis of ccRCC and offers a promising therapeutic target for the treatment of affected individuals.

### Supplementary Information

**Additional file 1: Figure S1.** Panoramic pictures of differentially expressed genes (DEGs). **A** Volcano plot of DEGs shows down-regulated DEGs in blue, up-regulated DEGs in red, and non-significant genes in grey (padj < 0.05, |log2FC|> 0). **B** Radar chart of the expression information for the top 15 upgrade and downgrade DEGs. The outermost numbers represent the log2FC value of each gene. The size of red and blue circles severally represent the log2FC value for up-regulated DEGs and down-regulated DEGs, and the higher the log2FC value, the bigger the circle. The red numbers and the blue numbers respectly forming a circle represent the expression value of each gene in High-CBX3 group and Low-CBX3 group. The connection of CBX3 and EMT-related genes such as VIM, CDH1 and CDH2 were performed. VIM (**C**) and CDH2 (**E**) were positively linked to CBX3 while CDH1 (**D**) was negatively associated with CBX3.

**Additional file 2: Figure S2.** Survival analysis of the different pathological stages: High CBX3 expression was linked to worse prognosis of T3 (**C**), N0 (**E**), NX (**G**) and M0 (**H**) (P < 0.05). CBX3 did not play a significant role in patient’s prognosis of the different pathological stages including T1 (**A**), T2 (**B**), T4 (**D**), N1 (**F**), M1 (**I**), MX (**J**), T1 + T2 (**K**).

**Additional file 3: Figure S3.** Correlation between CBX3 and significantly enriched genes in PI3K-Akt signaling pathway. CBX3 expression is positively correlated with **A** YWHAG (Pearson = 0.54), **B** CCNE2 (Pearson = 0.46), **C** CDK2 (Pearson = 0.41), **D** OSMR (Pearson = 0.41), **E** GNB4 (Pearson = 0.40), **F** MET (Pearson = 0.43), **G** IFNAR2 (Pearson = 0.45), **H** IL7 (Pearson = 0.49), **I** YWHAZ (Pearson = 0.44), **J** RAC1 (Pearson = 0.44), **K** RPS6KB1 (Pearson = 0.46) and **L** RHEB (Pearson = 0.44). However, CBX3 expression is negatively correlated with **M** GNG7 (Pearson = -0.49) and **K** ERBB2 (Pearson = − 0.47).

**Additional file 4: Figure S4.** Differences in the immune microenvironment scores between CBX3-high and CBX3-low groups. There was no significant difference in ESTIMATEScore (**A**), StromalScore (**B**), ImmuneScore (**C**) between the high and low CBX 3 expression groups.

**Additional file 5: Figure S5.** The correlation analysis between Immune cell infiltration and CBX3 expression. The bar plots showed the relative proportion of KIRC-infiltrating immune cells in groups with high CBX3 expression (**A**) and low CBX3 expression (**B**).

**Additional file 6: Figure S6.** The correlation analysis between PI3K-related genes and CBX3 expression with and without metastasis in ccRCC.

**Additional file 7: Figure S7.** The correlation analysis and the effects of PIK3R6 on immunity in KIRC. **A**, **B** The correlation analysis between PIK3R6 and CBX3 expression with and without metastasis in ccRCC. **C** The overall survival curve of PIK3R6 in ccRCC on the basis of the TCGA dataset. **D** PIK3R6 expression were associated with increases in score (EstimateScore, StromalScore, ImmuneScore) and decreases in tumor purity. **E** The correlation between the level of 24 infiltrating immune cells and PIK3R6 expression. *p < 0.05 is statistically significant.

## Data Availability

The original contributions presented in the study are included in the article/Supplementary Material. Further inquiries can be directed to the corresponding authors.

## Abbreviations

ccRCC: Clear cell renal cell carcinoma
EMT: Epithelial-to-mesenchymal transition
KIRC: Kidney renal clear cell carcinoma
CBX3: Chromobox homologue 3
CBX3-KD: CBX3 knockdown
DEGs: Differentially expressed genes
Padj: Adjusted p-value
KEGG: Kyoto Encyclopedia of Genes and Genomes
GO: GeneOntology
GSEA: Gene Set Enrichment Analysis
NES: Normalized enrichment score
PARP: Polymerase (ADP-ribose)
ICI: Immune checkpoint inhibitors
OS: Overall survival
ECAD: E-cadherin
VIM: Vimentin
NCAD: N-cadherin
pDCs: Plasmacytoid dendritic cells
DCs: Dendritic cells
P-AKT: Phosphorylated (p)-AKT
TME: Tumour microenvironment
TEBs: Tumor-educated B cells
