# Supplementary figures and images for: CBX3 promotes clear cell renal carcinoma through PI3K/AKT activation and aberrant immunity
Source: J Transl Med. 2023 Sep 6;21:600. doi: 10.1186/s12967-023-04478-9 (PMC10483741; doi:10.1186/s12967-023-04478-9)

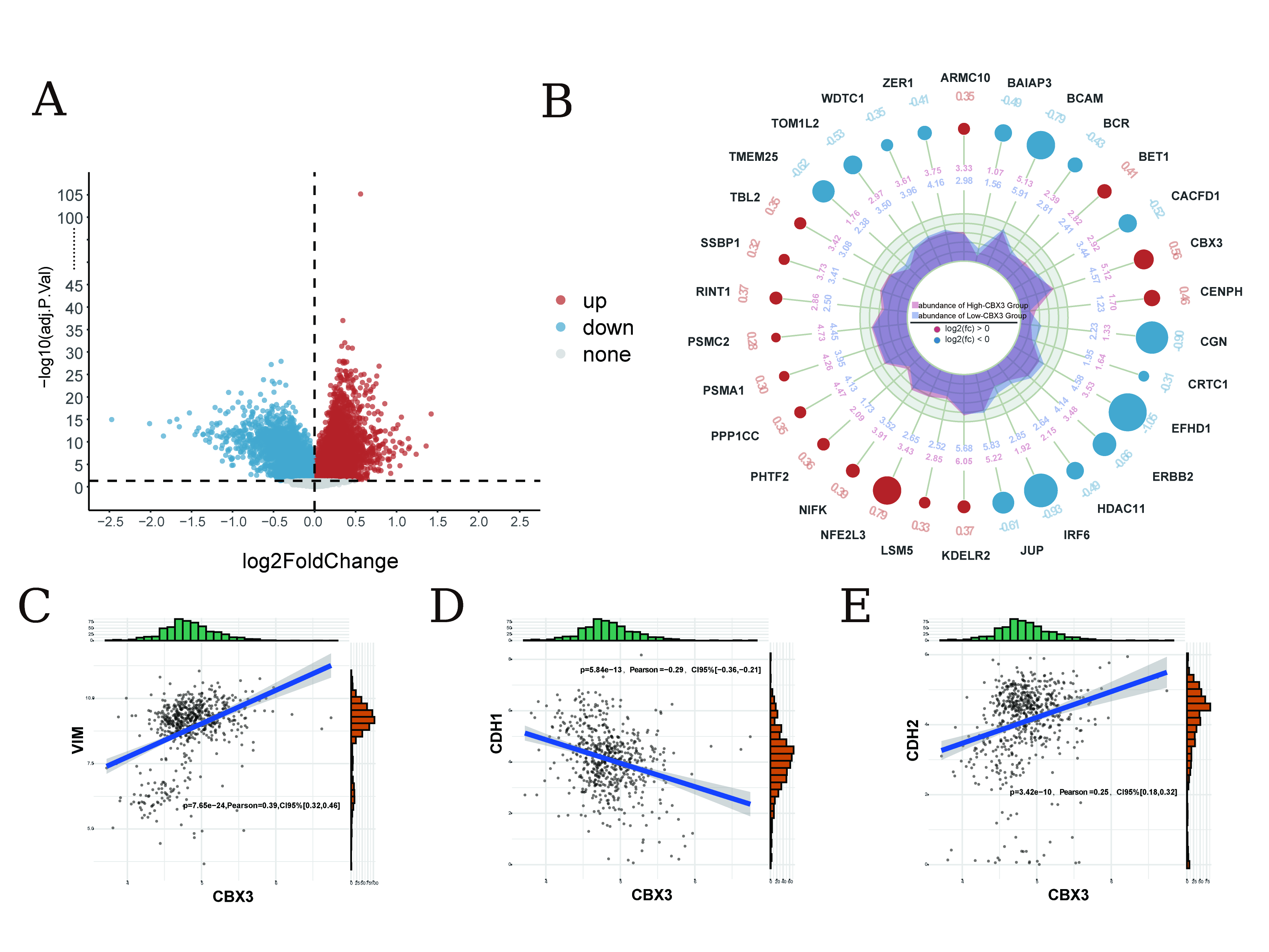

Supplement: Supplementary file 1 — Additional file 1: Figure S1. Panoramic pictures of differentially expressed genes (DEGs). A Volcano plot of DEGs shows down-regulated DEGs in blue, up-regulated DEGs in red, and non-significant genes in grey (padj < 0.05, |log2FC|> 0). B Radar chart of the expression information for the top 15 upgrade and downgrade DEGs. The outermost numbers represent the log2FC value of each gene. The size of red and blue circles severally represent the log2FC value for up-regulated DEGs and down-regulated DEGs, and the higher the log2FC value, the bigger the circle. The red numbers and the blue numbers respectly forming a circle represent the expression value of each gene in High-CBX3 group and Low-CBX3 group. The connection of CBX3 and EMT-related genes such as VIM, CDH1 and CDH2 were performed. VIM (C) and CDH2 (E) were positively linked to CBX3 while CDH1 (D) was negatively associated with CBX3. [file 12967_2023_4478_MOESM1_ESM.tif]

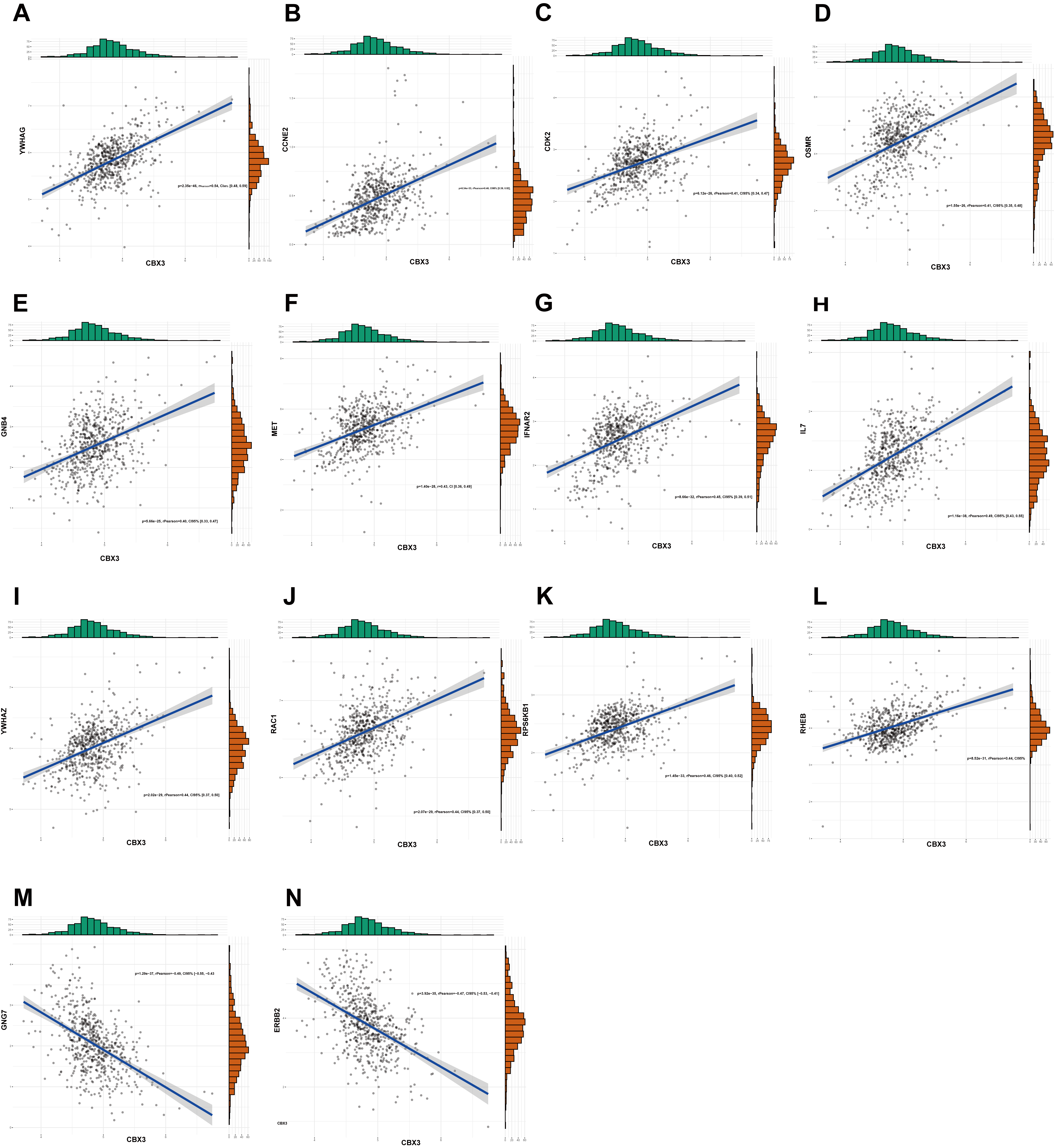

Supplement: Supplementary file 3 — Additional file 3: Figure S3. Correlation between CBX3 and significantly enriched genes in PI3K-Akt signaling pathway. CBX3 expression is positively correlated with A YWHAG (Pearson = 0.54), B CCNE2 (Pearson = 0.46), C CDK2 (Pearson = 0.41), D OSMR (Pearson = 0.41), E GNB4 (Pearson = 0.40), F MET (Pearson = 0.43), G IFNAR2 (Pearson = 0.45), H IL7 (Pearson = 0.49), I YWHAZ (Pearson = 0.44), J RAC1 (Pearson = 0.44), K RPS6KB1 (Pearson = 0.46) and L RHEB (Pearson = 0.44). However, CBX3 expression is negatively correlated with M GNG7 (Pearson = -0.49) and K ERBB2 (Pearson = − 0.47). [file 12967_2023_4478_MOESM3_ESM.tif]

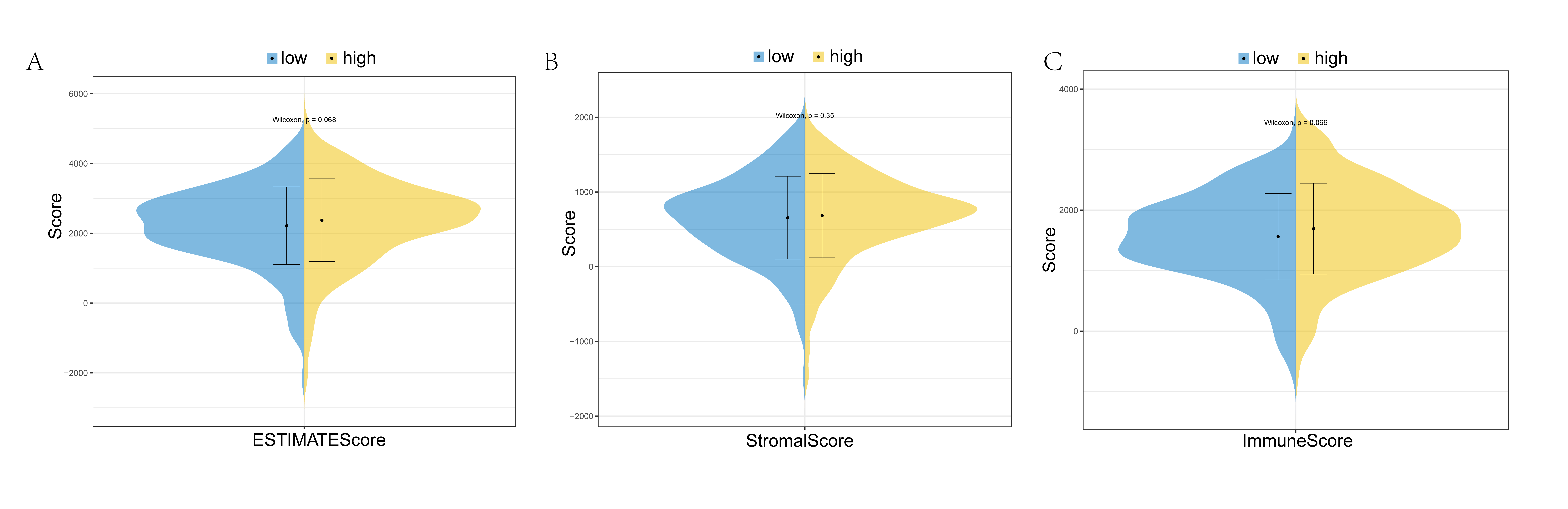

Supplement: Supplementary file 4 — Additional file 4: Figure S4. Differences in the immune microenvironment scores between CBX3-high and CBX3-low groups. There was no significant difference in ESTIMATEScore (A), StromalScore (B), ImmuneScore (C) between the high and low CBX 3 expression groups. [file 12967_2023_4478_MOESM4_ESM.tif]

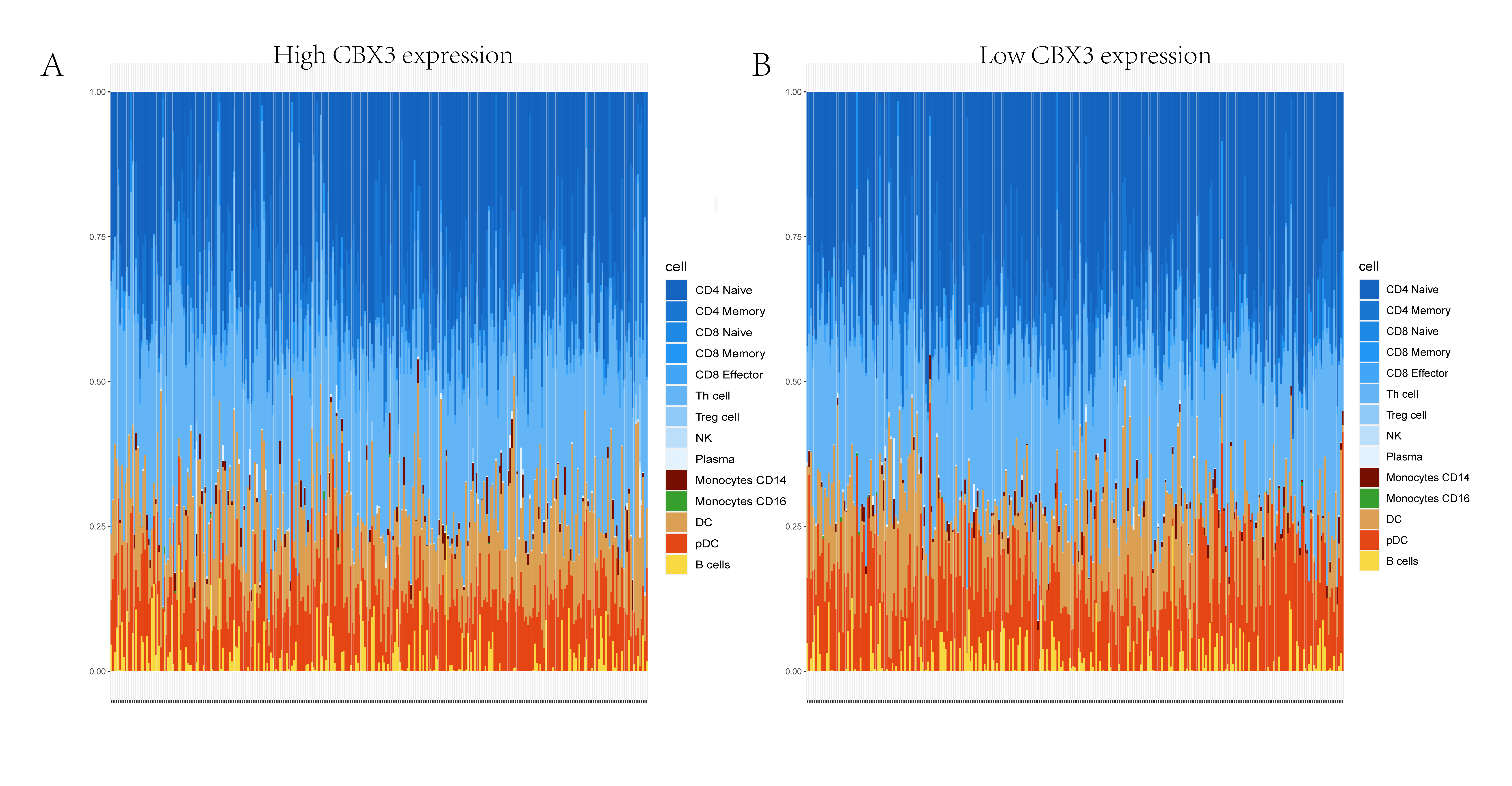

Supplement: Supplementary file 5 — Additional file 5: Figure S5. The correlation analysis between Immune cell infiltration and CBX3 expression. The bar plots showed the relative proportion of KIRC-infiltrating immune cells in groups with high CBX3 expression (A) and low CBX3 expression (B). [file 12967_2023_4478_MOESM5_ESM.tif]

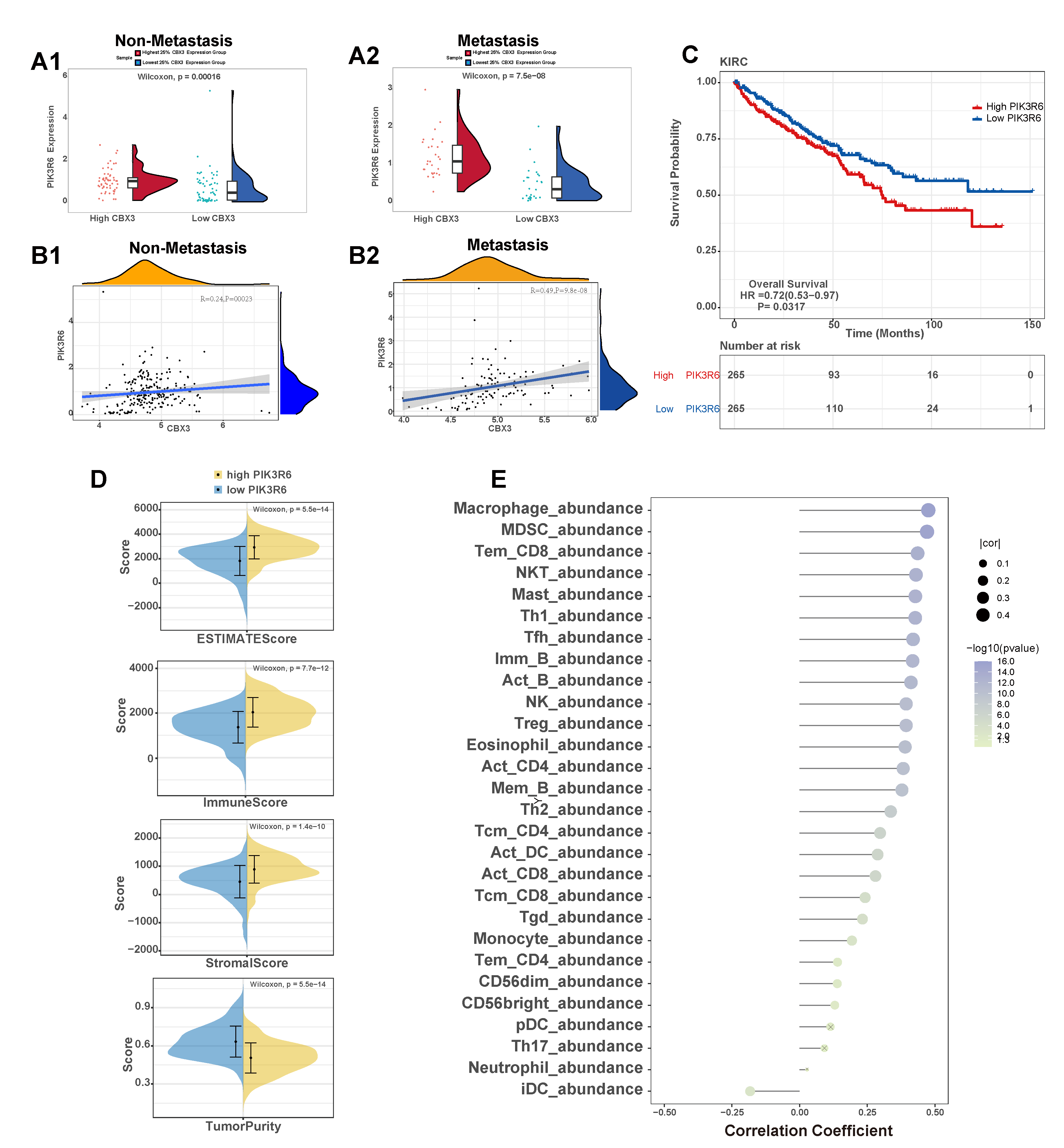

Supplement: Supplementary file 7 — Additional file 7: Figure S7. The correlation analysis and the effects of PIK3R6 on immunity in KIRC. A, B The correlation analysis between PIK3R6 and CBX3 expression with and without metastasis in ccRCC. C The overall survival curve of PIK3R6 in ccRCC on the basis of the TCGA dataset. D PIK3R6 expression were associated with increases in score (EstimateScore, StromalScore, ImmuneScore) and decreases in tumor purity. E The correlation between the level of 24 infiltrating immune cells and PIK3R6 expression. *p < 0.05 is statistically significant. [file 12967_2023_4478_MOESM7_ESM.tif]
